# Laparoscopic versus open resection for stage II/III rectal cancer in obese patients: A multicenter propensity score‐based analysis of short‐ and long‐term outcomes

**DOI:** 10.1002/ags3.12599

**Published:** 2022-07-16

**Authors:** Tomonori Akagi, Kentaro Nakajima, Yasumitsu Hirano, Tomoya Abe, Ryo Inada, Yohei Kono, Hidefumi Shiroshita, Tetsuji Ohyama, Masafumi Inomata, Seiichiro Yamamoto, Takeshi Naitoh, Yoshiharu Sakai, Masahiko Watanabe, the Japan Society of Laparoscopic Colorectal Surgery

**Affiliations:** ^1^ Gastroenterological and Pediatric Surgery Oita University of Faculty of Medicine Oita Japan; ^2^ Gastroenterological and Pediatric Surgery NTT Tokyo Japan; ^3^ Department of Gastroenterological Surgery Saitama Medical University International Medical Center Saitama Japan; ^4^ Department of Gastroenterological Surgery Sendai City Medical Center Sendai Open Hospital Sendai Japan; ^5^ Department of Gastroenterological Surgery Kochi Health Sciences Center Kochi Japan; ^6^ Biostatistics Center Kurume University Fukuoka Japan; ^7^ Department of Digestive Surgery Tokai University Hospital Isehara Japan; ^8^ Department of Lower Gastrointestinal Surgery Kitasato University School of Medicine Sagamihara Japan; ^9^ Department of Surgery Osaka Red Cross Hospital Osaka Japan; ^10^ Department of Surgery Kitasato University Kitasato Institute Hospital Tokyo Japan

**Keywords:** laparoscopic surgery, multicenter, obese, propensity score matching, rectal cancer

## Abstract

**Aim:**

Whether a laparoscopic procedure can contribute to the improvement of clinical outcomes in obese patients with stage II/III rectal cancer compared to an open procedure remains unclear.

**Objective:**

This study evaluated the technical and oncological safety of laparoscopic surgery versus open surgery in obese patients (body mass index [BMI] ≥25 kg/m^2^) with rectal cancer.

**Patients and Methods:**

Data were collected from patients with pathological stage II/III rectal cancer and analyzed. Operations were performed via laparoscopic or open surgery from 2009 to 2013. A comparative analysis was performed after applying propensity score matching to the two cohorts (laparoscopic group and open group). The primary endpoint was 3‐y relapse‐free survival (RFS).

**Results:**

Overall, 524 eligible cases were collected from 51 institutions. Equal numbers of propensity score‐matched patients were included in the laparoscopic (n = 193) group and open (n = 193) group. Although the rate of D3 lymph node dissection did not differ between the laparoscopic group (87.0%) and the open group (88.6%), the median number of harvested lymph nodes was significantly lower in the laparoscopic group versus open group (17.5 vs 21, *P* = 0.0047). The median postoperative hospital stay was also significantly shorter in the laparoscopic group (14 d) vs the open group (17 d) (*P* = 0.0014). Three‐y RFS was not significantly different between the two groups (hazard ratio 1.2454, 95% confidence interval 0.9201–1.6884, *P* = 0.4689).

**Conclusion:**

The short‐ and long‐term results of this large cohort study (UMIN ID: UMIN000033529) indicated that laparoscopic surgery in obese rectal cancer patients has advantageous short‐term outcomes and no disadvantageous long‐term outcomes.

## Graphical abstract

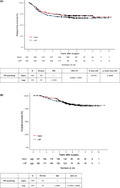

## 1 INTRODUCTION

Laparoscopic surgery for rectal cancer has been used worldwide, including in Japan, where it is performed not only for early‐stage rectal cancer but also for advanced rectal cancer.^1^, ^2^, ^3^ Several reports have compared short‐ and long‐term treatment outcomes of laparoscopic surgery for rectal cancer with those of open surgery. The safety and efficacy of laparoscopic surgery showed no significant differences in long‐term outcomes.^4^, ^5^, ^6^, ^7^ To date, some studies have reported laparoscopic surgery for obese patients with rectal cancer to be associated with fewer postoperative complications and less bleeding than with open surgery.^8^, ^9^ However, other studies found laparoscopic surgery for rectal cancer in obese patients to be technically more demanding than that in nonobese patients, and extra care was required to lessen the increased risk of developing postoperative complications.^10^, ^11^ Additionally, in the JCOG0404 randomized controlled trial (RCT), which examined survival outcomes following laparoscopic versus open D3 dissection for stage II/III colon cancer,^12^ among the obese colorectal cancer patients the prognosis in the laparoscopic surgery group was significantly poorer than that in the open surgery group.^13^ Although this trial showed the inferiority of laparoscopic surgery to open surgery in terms of long‐term outcomes in obese patients, almost no severely obese patients (with a body mass index [BMI] ≥30 kg/m^2^) were included, and thus, the type of recurrence could not be examined in detail due to the insufficient number of cases. In the present study, we focused on rectal cancer surgery, which is thought to be more difficult to perform than that for colon cancer, which was the subject of the JCOG0404 trial. There is a need for a larger‐scale analysis that evaluates the clinical outcomes of laparoscopic surgery for rectal cancer in severely obese patients.

Therefore, with use of the data of a large number of patients undergoing surgery at the facilities participating in the Japan Society of Laparoscopic Colorectal Surgery, we aimed to determine whether laparoscopic surgery for obese patients was noninferior to open surgery in terms of relapse‐free survival (RFS) using a propensity score (PS)‐matching analysis.

## 2 PATIENTS AND METHODS

This study involved patients with pathological stage II/III rectal cancer and BMI >25 kg/m^2^ who underwent open surgery or laparoscopic surgery at 51 institutions participating in the Japan Society of Laparoscopic Colorectal Surgery from January 2009 to December 2013. This cohort study and associated protocol were registered in UMIN in 2018 as UMIN000033529. After approval from each institutional Ethics Committee, patient data were collected from each clinical report form. Rectal cancer was defined as Ra, being the segment from the height of the inferior border of the second sacral vertebra to the peritoneal reflection, and as Rb, being the segment from the peritoneal reflection to the superior border of the puborectal sling. Eligibility criteria were (a) pathological stage II/III rectal cancer with curative resection, (b) BMI ≥25 kg/m^2^, and (c) age 20–79 y old. The exclusion criteria were (i) active multiple malignancies at diagnosis, (ii) cancer of the appendix, (iii) previous intestinal resection surgery (excluding appendectomy), or (iv) multiple colorectal cancers. The demographic and clinicopathological data of consecutive patients were collected retrospectively and included age, sex, BMI, comorbidity, pathological depth of tumor invasion, pathological lymph node metastasis, lesion locale, estimated blood loss (EBL), operation time, degree of lymph node dissection, lymph nodes retrieved, lymph node metastases, postoperative complications, 30‐d mortality, length of hospital stay, first recurrent organ, and RFS and overall survival (OS) periods.

The primary endpoint of this study was RFS. The secondary endpoints were OS, EBL, operation time, the degree of lymph node dissection, lymph nodes retrieved, lymph node metastases, postoperative complications, 30‐d mortality, length of hospital stay, and first recurrent organ.

The required sample size of 802 in each group was estimated based on a one‐sided significance level of 5% and power of 80%, assuming 3‐y RFS in open surgery to be 72% and a noninferiority margin of −6%, which would yield a noninferiority margin hazard ratio (HR) of 1.2645.

### 2.1 Statistical analysis

Case matching was performed with the PS estimated by a logistic regression model with the following 12 factors: age; sex; BMI; history of the comorbidities of hypertension, diabetes mellitus, cerebrovascular disease, respiratory disease, and cardiovascular disease; tumor location; depth of tumor invasion; lymph node metastasis; and creation of a stoma. Nearest‐neighbor matching without replacement within a caliper was used. The size of the caliper was set at 0.2 of the standard deviation of the logit of the estimated PS. Patients who were found to be outside the caliper were excluded, as were unmatched patients. We assessed the degree of imbalance of patient characteristics using the absolute standardized difference (*d*). We considered small (<10) values of *d* to support the assumption of balance between groups. All statistical analyses for primary and secondary endpoints were performed on all matched‐pair patients. OS was calculated from the date of operation until death from any cause or the date of the last follow‐up. RFS was calculated from the date of the operation until the date of confirmed recurrence or any cause of death. Survival curves were estimated using the Kaplan–Meier method, and HRs were estimated by the Cox regression model with factors having the value of *d* after PS matching greater than 10. Categorical variables were analyzed with Fisher's exact test, and continuous variables were analyzed using the Wilcoxon test. We used a one‐sided significance level of 0.05 in the primary analysis. All *P* values in the secondary analyses were two‐sided, and values <0.05 were considered statistically significant. PS matching and all other analyses were performed with R software v. 4.02 (R Core Team, Vienna, Austria).

## 3 RESULTS

**FIGURE 1:**
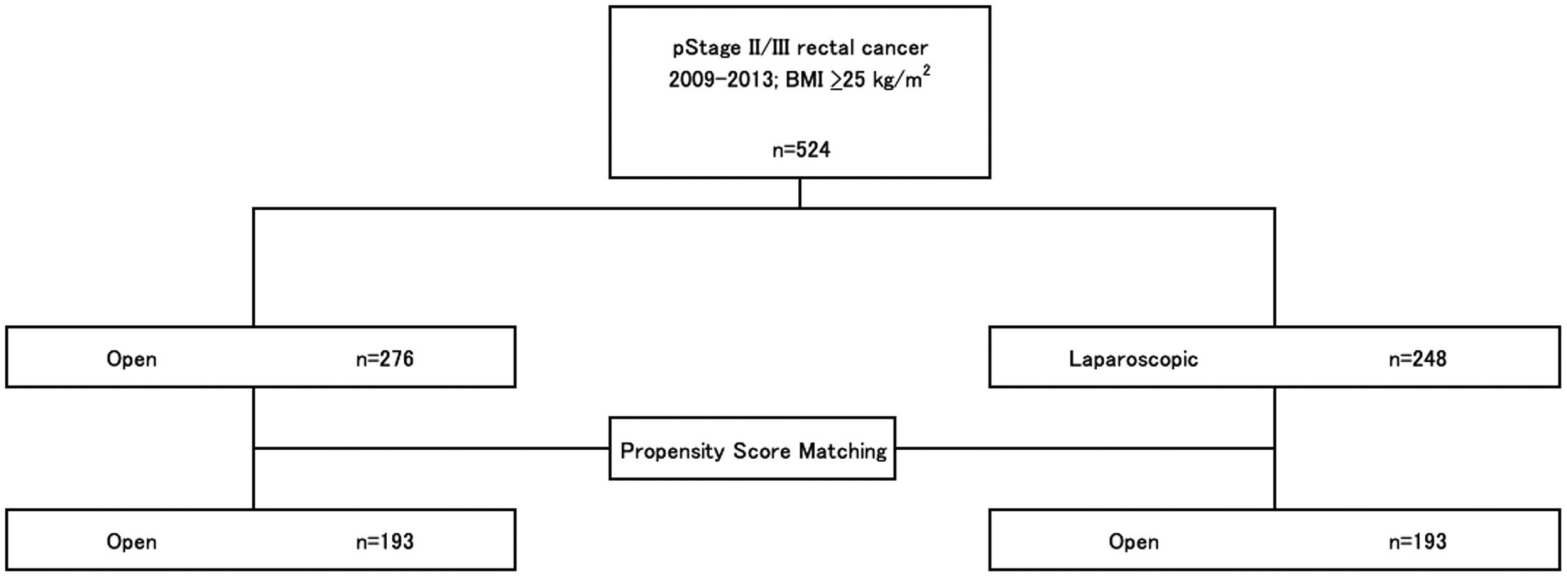
Flowchart of patient inclusion and exclusion and number of patients operated on. ASA, American Society of Anesthesiologists; BMI, body mass index

Data from 524 patients were collected from 51 institutions. Among these 524 patients, open surgery was performed in 276 and laparoscopic surgery was performed in 248. Seven patients were excluded from all registered patients because their RFS data were missing. PS matching for the long‐term endpoint analyses was conducted, and after matching, 193 pairs of patients were respectively analyzed for the long‐ and short‐term endpoints (Figure 1). The median follow‐up period was 5.6 y.

Table 1 summarizes the background data of the overall cohort and matched cases. Before matching, a history of hypertension and diabetes mellitus was often observed in the open group. Pathological depth of tumor invasion was also deeper in the open group. After matching, all patient characteristics were balanced except for a history of hypertension and pathological lymph node metastasis. The mean BMI was 27.5 kg/m^2^ in the open group and 27.5 kg/m^2^ in the laparoscopic group (Table 1).

**TABLE 1:** Patient and tumor characteristics

	Before PS matching			After PS matching		
	Open group (N = 274)	Laparoscopic group (N = 243)	*d*	Open group (N = 193)	Laparoscopic group (N = 193)	*d*
Age, y, mean (SD)	61.4 (9.7)	61.6 (10.1)	2.74	61.4 (9.2)	61.8 (10.2)	3.24
Sex						
Female	86 (31.4%)	67 (27.6%)	8.36	54 (28.0%)	54 (28.0%)	0.00
Male	188 (68.6%)	176 (72.4%)		139 (72.0%)	139 (72.0%)	
BMI, kg/m^2^, mean (SD)	27.5 (2.4)	27.5 (2.3)	1.88	27.5 (2.5)	27.6 (2.2)	4.16
Hypertension						
No	151 (55.1%)	153 (63.0%)	15.99	109 (56.5%)	124 (64.2%)	15.90
Yes	123 (44.9%)	90 (37.0%)		84 (43.5%)	69 (35.8%)	
Diabetes mellitus						
No	219 (79.9%)	202 (83.1%)	8.24	159 (82.4%)	159 (82.4%)	0.00
Yes	55 (20.1%)	41 (16.9%)		34 (17.6%)	34 (17.6%)	
Cerebrovascular disease						
No	267 (97.4%)	232 (95.5%)	10.66	186 (96.4%)	184 (95.3%)	5.19
Yes	7 (2.6%)	11 (4.5%)		7 (3.6%)	9 (4.7%)	
Respiratory disease						
No	263 (96.0%)	232 (95.5%)	2.53	186 (96.4%)	183 (94.8%)	7.56
Yes	11 (4.0%)	11 (4.5%)		7 (3.6%)	10 (5.2%)	
Cardiovascular diseases						
No	255 (93.1%)	222 (91.4%)	6.36	180 (93.3%)	177 (91.7%)	5.88
Yes	19 (6.9%)	21 (8.6%)		13 (6.7%)	16 (8.3%)	
Tumor location						
Ra	102 (37.2%)	142 (58.4%)	43.37	102 (52.8%)	101 (52.3%)	1.03
Rb	172 (62.8%)	101 (41.6%)		91 (47.2%)	92 (47.7%)	
Pathological depth						
T1/T2	35 (12.8%)	49 (20.2%)	19.99	33 (17.1%)	32 (16.6%)	1.38
T3/T4a/T4b	239 (87.2%)	194 (79.8%)		160 (82.9%)	161 (83.4%)	
Pathological lymph node metastasis						
N0	107 (39.1%)	101 (41.6%)	5.11	74 (38.3%)	85 (44.0%)	11.57
N1/N2/N3	167 (60.9%)	142 (58.4%)		119 (61.7%)	108 (56.0%)	
Stoma creation						
No	267 (97.4%)	221 (90.9%)	28.01	186 (96.4%)	183 (94.8%)	7.56
Yes	7 (2.6%)	22 (9.1%)		7 (3.6%)	10 (5.2%)	

*Note*: Absolute standardized difference *d* is defined by $100\times \left|{\bar{x}}_{1}-{\bar{x}}_{2}\right|/\sqrt{\left(s_{1}^{2}+s_{2}^{2}\right)/2}$, where ${\bar{x}}_{1},{\bar{x}}_{2}$ are group means, and $s_{1}^{2},s_{2}^{2}$ are group variances.

Abbreviations: BMI, body mass index; PS, propensity score; SD, standard deviation.

The operative outcomes are summarized in Table 2. Intraoperative EBL was significantly lower in the laparoscopic group (50 mL) than that in the open group (480 mL) (*P* < 0.0001). The operative time was significantly shorter in the open group (293 min) than that in the laparoscopic group (323 min) (*P* = 0.075). The proportion of D3 lymph node dissection was not different between the laparoscopic group (87.0%) and the open group (88.6%). Although the number of retrieved lymph nodes was significantly lower in the laparoscopic group than that in the open group, the number of lymph node metastases was not different. Table 3 shows the operative outcomes based on the presence or absence of lateral lymph node dissection. Comparison between the two groups after matching showed that the laparoscopic group experienced significantly less blood loss and longer operative times than the open group, with or without lateral dissection. Further, after matching, the number of dissected nodes was significantly lower in the laparoscopic group compared to the open group in the patients who underwent lateral dissection, but there was no difference in the number of metastatic lymph nodes. There was also no difference in the number of dissected and metastatic lymph nodes in the patients who did not undergo lateral dissection compared to those who underwent it in the laparoscopic and open groups. Short‐term outcomes and postoperative complications (greater than Clavien–Dindo grade 3) including anastomotic leakage showed no statistical difference between the groups (Table 4). No instance of 30‐d mortality was observed in either group. The median postoperative hospital stay was significantly shorter in the laparoscopic group (14 d) than that in the open group (17 d) (*P* = 0.0014).

**TABLE 2:** Operative outcomes

	Before PS matching			After PS matching		
	Open group (N = 274)	Laparoscopic group (N = 243)	*P* value	Open group (N = 193)	Laparoscopic group (N = 193)	*P* value
EBL, mL						
Median	522.5	45	<0.0001	480	50	<0.0001
IQR	256.2, 1064.5	10.0, 175.0		215.0, 990.0	15.0, 200.0	
Range	10.0–4900.0	0.0–2080.0		10.0–4900.0	0.0–2080.0	
Operation time, min						
Median	308	311	0.6849	293	323	0.0075
IQR	230.0, 404.5	245.0, 410.0		211.0, 379.0	245.0, 423.0	
Range	123.0–882.0	100.0–915.0		123.0–810.0	100.0–915.0	
Degree of LN dissection						
D0	1 (0.4%)	0 (0.0%)	0.4067	1 (0.5%)	0 (0.0%)	0.6378
D1	0 (0.0%)	0 (0.0%)		0 (0.0%)	0 (0.0%)	
D2	28 (10.2%)	31 (12.8%)		21 (10.9%)	25 (13.0%)	
D3	245 (89.4%)	212 (87.2%)		171 (88.6%)	168 (87.0%)	
LNs retrieved, n						
Median	23	18	<0.0001	21	17.5	0.0047
IQR	14.0, 34.0	12.0, 27.0		12.0, 32.0	12.0, 26.2	
Range	0.0–145.0	0.0–109.0		0.0–110.0	0.0–109.0	
Missing	0	1		0	1	
LN metastases, n						
Median	1	1	0.2858	1	1	0.2560
IQR	0.0, 3.0	0.0, 3.0		0.0, 3.0	0.0, 3.0	
Range	0.0–39.0	0.0–22.0		0.0–39.0	0.0–22.0	
Procedure, n						
HAR	6 (2.2%)	4 (1.6%)	<0.0001	6 (3.1%)	3 (1.6%)	0.0528
LAR (ISR)	182 (66.4%)	216 (88.9%)		146 (75.6%)	167 (86.5%)	
APR	79 (28.8%)	19 (7.8%)		37 (19.2%)	19 (9.8%)	
Hartmann	4 (1.5%)	3 (1.2%)		3 (1.6%)	3 (1.6%)	
Missing	3 (1.1%)	1 (0.4%)		1 (0.5%)	1 (0.5%)	
Pelvic lateral LN dissection						
No	168 (62.0%)	200 (82.3%)	<0.0001	130 (68.4%)	153 (79.3%)	0.0198
Yes	103 (38.0%)	43 (17.7%)		60 (31.6%)	40 (20.7%)	
Missing	3	0		3	0	

Abbreviations: APR, abdominoperineal resection; EBL, estimated blood loss; HAR, high anterior resection; IQR, interquartile range; ISR, intersphincteric resection; LAR, low anterior resection; LN, lymph node; PS, propensity score.

**TABLE 3:** Operative outcomes with or without lateral dissection

	LLND		Before PS matching			After PS matching		
			Open group	Laparoscopic group	*P* value	Open group	Laparoscopic group	*P* value
EBL, mL	No	N	168	200	<0.0001	130	153	<0.0001
		Median	415	30		390	35	
		IQR	200.0, 802.5	10.0, 141.2		190.0, 740.0	10.0, 150.0	
		Range	10.0–3439.0	0.0–1415.0		10.0–2320.0	0.0–1415.0	
	Yes	N	103	43	<0.0001	60	40	<0.0001
		Median	880	200		992.5	300	
		IQR	490.0, 1322.5	88.5, 500.0		495.0, 1332.5	95.8, 542.5	
		Range	10.0–4900.0	0.0–2080.0		10.0–4900.0	0.0–2080.0	
Operation time, min	No	N	168	200	0.0166	130	153	0.0003
		Median	265.5	285.5		246	296	
		IQR	201.5, 342.5	235.0, 365.5		192.2, 327.8	235.0, 380.0	
		Range	123.0–810.0	100.0–762.0		123.0–810.0	100.0–762.0	
	Yes	N	103	43	0.0318	60	40	0.0308
		Median	392	455		385.5	461.5	
		IQR	310.0, 488.5	376.0, 495.5		310.0, 486.2	378.2, 499.0	
		Range	148.0–882.0	230.0–915.0		148.0–702.0	230.0–915.0	
LNs retrieved, n	No	N	168	199	0.1110	130	153	0.2064
		Median	19	16		17.5	15.5	
		IQR	11.0, 25.5	11.0, 24.0		11.0, 26.5	10.8, 23.0	
		Range	0.0–145.0	0.0–75.0		0.0–81.0	0.0–75.0	
		Missing	0	1		0	1	
	Yes	N	103	43	0.0304	60	40	0.0266
		Median	33	27		33	25	
		IQR	23.5, 45.0	16.5, 36.5		22.8, 49.2	16.8, 36.0	
		Range	3.0–110.0	0.0–109.0		7.0–110.0	0.0–109.0	
LN metastases, n	No	N	168	200	0.7311	130	153	0.3344
		Median	1	1		1	1	
		IQR	0.0, 3.0	0.0, 2.0		0.0, 3.0	0.0, 2.0	
		Range	0.0–27.0	0.0–22.0		0.0–27.0	0.0–22.0	
	Yes	N	103	43	0.6378	60	40	
		Median	1	1		1	1	0.9595
		IQR	0.0, 3.0	0.0, 3.0		0.0, 3.0	0.0, 3.5	
		Range	0.0–39.0	0.0–10.0		0.0–39.0	0.0–10.0	

Abbreviations: EBL, estimated blood loss; IQR, interquartile range; LLND, lateral lymph node dissection; LN, lymph node; PS, propensity score.

**TABLE 4:** Short‐term outcomes

	Before PS matching			After PS matching		
	Open group (N = 274)	Laparoscopic group (N = 243)	*P* value	Open group (N = 193)	Laparoscopic group (N = 193)	*P* value
Complication ≥C‐D grade 3 (Overall)						
No	229 (83.6%)	206 (84.8%)	0.7194	165 (85.5%)	163 (84.5%)	0.8868
Yes	45 (16.4%)	37 (15.2%)		28 (14.5%)	30 (15.5%)	
Anastomotic leakage						
No	255 (93.4%)	220 (90.5%)	0.2557	179 (93.2%)	176 (91.2%)	0.5691
Yes	18 (6.6%)	23 (9.5%)		13 (6.8%)	17 (8.8%)	
Missing	1	0		1	0	
Ileus (overall)						
No	268 (97.8%)	238 (97.9%)	1	187 (96.9%)	189 (97.9%)	0.7507
Yes	6 (2.2%)	5 (2.1%)		6 (3.1%)	4 (2.1%)	
Obstructive ileus						
No	272 (99.3%)	239 (98.4%)	0.4269	191 (99.0%)	190 (98.4%)	1
Yes	2 (0.7%)	4 (1.6%)		2 (1.0%)	3 (1.6%)	
Paralytic ileus						
No	270 (98.5%)	242 (99.6%)	0.3771	189 (97.9%)	192 (99.5%)	0.3717
Yes	4 (1.5%)	1 (0.4%)		4 (2.1%)	1 (0.5%)	
30‐d mortality						
None	273 (99.6%)	243 (100.0%)	1	193 (100.0%)	193 (100.0%)	1
Missing	1 (0.4%)	0 (0.0%)		0 (0.0%)	0 (0.0%)	
Length of hospital stay, d						
Median	19	14	<0.0001	17	14	0.0014
IQR	14.0, 28.0	10.0, 22.0		13.0, 26.0	10.0, 23.0	
Range	0.0–67.0	6.0–86.0	0.0–67.0	6.0–86.0

Abbreviations: C‐D, Clavien–Dindo; IQR, interquartile range; PS, propensity score.

**FIGURE 2:**
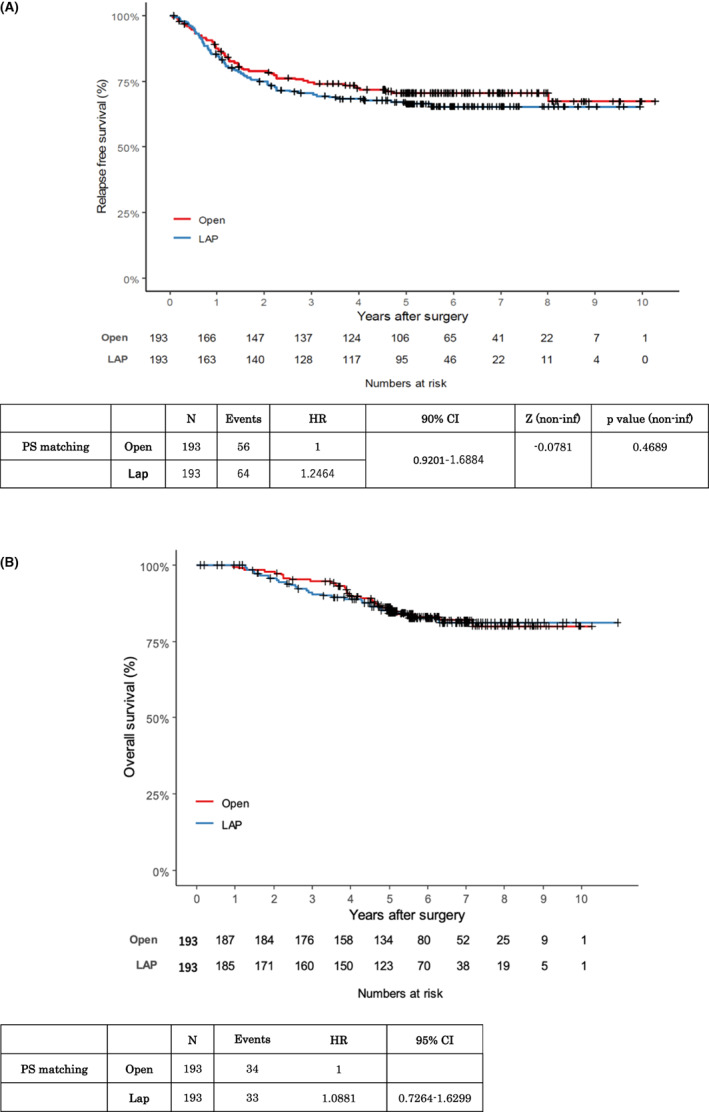
Kaplan–Meier plots of patient survival. A: Kaplan–Meier plot for relapse‐free survival (RFS) after propensity score (PS) matching. B: Kaplan–Meier plot for overall survival (OS) after PS matching. CI, confidence interval; HR, hazard ratio; LAP, laparoscopic group; open, open group

RFS did not differ between the two groups (Figure 2A). After matching, the 3‐y estimated rates of RFS for patients in the open group and laparoscopic group were 74.6% and 70.5%, respectively. Cox regression analysis was performed with two adjustment factors, a history of hypertension and lymph node metastasis, because their *d* values were greater than 10 after matching. The adjusted HR for RFS for laparoscopic surgery versus open surgery was 1.2464 (90% confidence interval [CI]: 0.9201–1.6884, one‐sided *P* for noninferiority: 0.4689), which did not indicate that laparoscopic surgery for obese patients with rectal cancer was noninferior to open surgery due to a shortage of events. The rate of OS was analyzed and was also found not to differ between the two groups (Figure 2B). After matching, the 3‐y estimated rates of OS for patients in the open group and laparoscopic group were 93.6% and 91.2%, respectively. The adjusted HR for OS for laparoscopic surgery versus open surgery was 1.0881 (95% CI 0.7264–1.6299). There were no differences in the sites of recurrence between the laparoscopic and open surgery groups (Table S1).

## 4 DISCUSSION

Using a large‐scale PS‐matching analysis, the present study could not statistically show laparoscopic surgery for obese patients (BMI ≥25 kg/m^2^) with rectal cancer to be noninferior to open surgery with regard to long‐term outcome. However, because the RFS in both groups was almost similar, we concluded that laparoscopic surgery offers advantages for short‐term outcomes and no disadvantages for long‐term outcomes, and the use of laparoscopic procedures to treat stage II/III advanced rectal cancer in obese patients appears to be acceptable. Although the Japanese guideline recommends careful selection of obese patients with colorectal cancer for treatment by laparoscopic surgery,^14^ laparoscopic surgery for rectal cancer has been spreading drastically in Japan.^3^ Presently, however, the evidence is insufficient for performing laparoscopic resection of rectal cancer in obese patients. To our knowledge, the present study is the largest study to date to focus on the treatment of stage II/III rectal cancer in obese patients with a BMI ≥25 kg/m^2^.

We performed PS matching to balance the background patient data. Twelve factors were used, as described in the protocol and the Methods sections. Matching resulted in patient background factors that were ideally balanced between the groups, and thus, we considered the comparison between open surgery and laparoscopic surgery to be reliable.

Our data showed that the 3‐y rate of RFS after laparoscopic surgery was not noninferior to that of open surgery. However, RFS was almost similar in both groups. Despite not being noninferior, because the RFS in both groups was similar, laparoscopic D3 dissection seems to be technically and oncologically feasible for stages II or III rectal cancer in obese patients. To the best of our knowledge, this is also the first large‐scale PS‐matching analysis focused on advanced rectal cancer in obese patients to assess long‐term outcomes. One possible explanation for why noninferiority was not shown was that the number of cases collected was less than expected in both groups, despite revision of the protocol and extension of the enrollment period from 3 to 5 y. The finding in the present study of better short‐term outcomes in laparoscopic surgery without affecting long‐term outcome compared to open surgery was consistent with that of previous reports.^4^, ^5^, ^6^, ^7^ We are fully aware that the number of events involved in the long‐term outcome of the present study was insufficient, and thus, further study will be required. Although it would be difficult at present to conduct an RCT comparing the oncological feasibility of laparoscopic surgery to that of open surgery in obese patients, the present large‐scale PS‐matched analysis yielded reliable evidence for the current clinical practice. The present study also showed that the proportion and site of recurrence in the patients with a higher BMI were not apparently different between open surgery and laparoscopic surgery.

Body mass index is commonly used as an objective measure of body fat. The World Health Organization has determined a global cutoff point for obesity to be **≥**30.0 kg/m^2^.^15^ However, in the JCOG0404 trial the BMI was **≥**30.0 kg/m^2^ in only 2.3% of the obese patients. Despite the average BMI being lower in Asian populations than in non‐Asian populations, the rate of visceral adiposity is higher in Asians.^16^ Because this cutoff point is inappropriate for the Asian population, the International Obesity Task Force has proposed a lower cutoff value for BMI of **≥**25.0 kg/m^2^ to indicate obesity in Asians,^15^, ^16^, ^17^ and this value was used in the present study.

Among the short‐term outcomes in obese patients, the laparoscopic group had significantly less intraoperative blood loss, shorter postoperative stay, fewer dissected lymph nodes, and longer operative time than those of the open group. The shorter length of postoperative stay shown in our data from the laparoscopic group was consistent with the outcome from JCOG0404. In terms of numbers of lymph nodes dissected, fewer were dissected in the laparoscopic group. When an anastomosis is planned in laparoscopic surgery, the proximal resection length is limited by the tension‐free anastomosis. In open surgery, however, anastomotic tension can be easily checked before the anastomosis is created, so proximal additional resection is often performed beyond the oncological safety margin. This might account for the difference in the number of resected lymph nodes between the two groups. In previous reports, laparoscopic surgery resulted in shorter proximal margins and a tendency for fewer lymph nodes to be dissected than those in open surgery.^5^, ^6^ In addition, the proportion of lateral lymph node dissection was higher in the open group than in the laparoscopic group, which was considered to be the reason.

Our study has several strengths, including the number of patients analyzed, the large number of participating institutions, the meticulous selection of only patients with higher BMI and advanced rectal cancer requiring D3 lymph node dissection, and the validated populations matched by PS. In addition, the period between 2009 and 2013 during which the patients underwent rectal cancer surgery was almost aligned with current clinical practice compared to that during the period between 2004 and 2014 in JCOG0404. Although some limitations exist, such as selection bias due to the retrospective cohort in our study, this was reduced as much as possible by using PS matching. Total mesocolic excision is known to be an important procedure, but its quality was not assessed in this study. Because conceivable bias was reduced as much as possible by the use of PS matching, we thus believe that the present study offers the highest level of evidence currently available on the validity of performing laparoscopic surgery in obese patients with stage II/III rectal cancer.

## 5 CONCLUSIONS

Laparoscopic surgeries in obese patients with stage II/III rectal cancer were safely performed in the 51 participating institutions. In obese patients, laparoscopic surgery was more useful than open surgery due to the shorter postoperative stay following laparoscopic surgery. Further, the rates of RFS and OS were not significantly different between the laparoscopic and open surgery groups in this study. Thus, even for obese patients with advanced rectal cancer, laparoscopic surgery may be considered a useful option based on the results of this large cohort study.

## DISCLOSURE

Funding: This work was supported by grants from the Japan Society of Clinical Oncology and the Japanese Foundation for Research and Promotion of Endoscopy.

Conflict of Interest: Masafumi Inomata is an Editorial Board Member of the *Annals of Gastroenterological Surgery* dealing with the lower digestive tract. All of the other authors declare no conflicts of interest.

Author Contributions: Tomonori Akagi, Kentaro Nakajima, Yohei Kono, Hidefumi Shiroshita, Masafumi Inomata, Seiichiro Yamamoto, Takeshi Naitoh, Yoshiharu Sakai, and Masahiko Watanabe contributed to the study design. Yasumitsu Hirano, Ryo Inada, Tomonori Akagi, Yohei Kono, and Seiichiro Yamamoto contributed to data collection, data analysis, and interpretation. Tetsuji Ohyama contributed to data management, statistical analysis, and data interpretation. All the authors contributed to writing or reviewing the report and approved the final version.

## APPROVAL OF THE RESEARCH PROTOCOL

After approval from each institutional Ethics Committee, patient data were collected from each clinical report form.

## REGISTRY AND THE REGISTRATION NO. OF THE STUDY/TRIAL

This cohort study and associated protocol were registered in UMIN in 2018 (UMIN000033529).

## Supporting information


Table S1
