# Supplementary material for: Laparoscopic versus open resection for stage II/III rectal cancer in obese patients: A multicenter propensity score‐based analysis of short‐ and long‐term outcomes
Source: Ann Gastroenterol Surg. 2022 Jul 16;7(1):71–80. doi: 10.1002/ags3.12599 (PMC9831897; doi:10.1002/ags3.12599)
Supplement: Supplementary file 1 — Table S1 [file AGS3-7-71-s001.docx]

| **SUPPLEMENTARY TABLE 1.** First site of recurrence | | | | | | |
| --- | --- | --- | --- | --- | --- | --- |
|  | Before PS matching | |  | After PS matching | |  |
|  | Open group (N=274) | Laparoscopic group (N=243) | p Value | Open group (N=193) | Laparoscopic group  (N=193) | p Value |
| First recurrent organ |  |  |  |  |  |  |
| Locoregional (Anastomotic site） | |  | 0.3368 |  |  | 0.2198 |
| No | 262 (95.6%) | 237 (97.5%) |  | 185 (95.9%) | 190 (98.4%) |  |
| Yes | 12 (4.4%) | 6 (2.5%) |  | 8 (4.1%) | 3 (1.6%) |  |
| Locoregional (Regional lymph nodes) | |  | 0.1830 |  |  | 0.4488 |
| No | 267 (97.4%) | 241 (99.2%) |  | 188 (97.4%) | 191 (99.0%) |  |
| Yes | 7 (2.6%) | 2 (0.8%) |  | 5 (2.6%) | 2 (1.0%) |  |
| Locoregional (Other) |  |  | 0.6833 |  |  | 0.3470 |
| No | 262 (95.6%) | 230 (94.7%) |  | 186 (96.4%) | 181 (93.8%) |  |
| Yes | 12 (4.4%) | 13 (5.3%) |  | 7 (3.6%) | 12 (6.2%) |  |
| Distant lymph node metastasis | |  | 0.6644 |  |  | 1 |
| No | 261 (95.3%) | 234 (96.3%) |  | 185 (95.9%) | 184 (95.3%) |  |
| Yes | 13 (4.7%) | 9 (3.7%) |  | 8 (4.1%) | 9 (4.7%) |  |
| Liver |  |  | 0.1212 |  |  | 0.0827 |
| No | 255 (93.1%) | 216 (88.9%) |  | 180 (93.3%) | 169 (87.6%) |  |
| Yes | 19 (6.9%) | 27 (11.1%) |  | 13 (6.7%) | 24 (12.4%) |  |
| Lung |  |  | 0.8806 |  |  | 0.7466 |
| No | 249 (90.9%) | 219 (90.1%) |  | 173 (89.6%) | 170 (88.1%) |  |
| Yes | 25 (9.1%) | 24 (9.9%) |  | 20 (10.4%) | 23 (11.9%) |  |
| Hemostatic metastasis |  |  | 1 |  |  | 1 |
| No | 273 (99.6%) | 243 (100.0%) |  | 192 (99.5%) | 193 (100.0%) |  |
| Yes | 1 (0.4%) | 0 (0.0%) |  | 1 (0.5%) | 0 (0.0%) |  |
| Peritoneal |  |  | 0.1922 |  |  | 0.1231 |
| No | 273 (99.6%) | 239 (98.4%) |  | 193 (100.0%) | 189 (97.6%) |  |
| Yes | 1 (0.4%) | 4 (1.6%) |  | 0 (0.0%) | 4 (2.1%) |  |
| Bone |  |  | 1 |  |  | 1 |
| No | 270 (98.5%) | 240 (98.8%) |  | 190 (98.4%) | 191 (99.0%) |  |
| Yes | 4 (1.5%) | 3 (1.2%) |  | 3 (1.6%) | 2 (1.0%) |  |
| Adrenal |  |  | 1 |  |  | 1 |
| No | 272 (99.3%) | 242 (99.6%) |  | 192 (99.5%) | 193 (100.0%) |  |
| Yes | 2 (0.7%) | 1 (0.4%) |  | 1 (0.5%) | 0 (0.0%) |  |
| Brain |  |  | 0.2204 |  |  | 0.4987 |
| No | 274 (100.0%) | 241 (99.2%) |  | 193 (100.0%) | 191 (99.0%) |  |
| Yes | 0 (0.0%) | 2 (0.8%) |  | 0 (0.0%) | 2 (1.0%) |  |
| Skin |  |  |  |  |  |  |
| No | 274 (100.0%) | 243 (100.0%) |  | 193 (100.0%) | 193 (100.0%) |  |
| Ovary |  |  |  |  |  |  |
| No | 274 (100.0%) | 243 (100.0%) |  | 193 (100.0%) | 193 (100.0%) |  |
| Port site |  |  |  |  |  |  |
| No | 274 (100.0%) | 243 (100.0%) |  | 193 (100.0%) | 193 (100.0%) |  |
| Abbreviation: PS, propensity score. | | | | | | |
